# Displacement Measurement Method Based on Double-Arrowhead Auxetic Tubular Structure

**DOI:** 10.3390/s23239544

**Published:** 2023-11-30

**Authors:** Qingguo Wen, Pengju Li, Zhengkai Zhang, Hong Hu

**Affiliations:** 1 School of Mechanical and Electrical Engineering, Xi’an University of Architecture and Technology, Xi’an 710055, China; wenqingguo@xauat.edu.cn (Q.W.); engineer53@163.com (P.L.); 2 School of Fashion and Textiles, The Hong Kong Polytechnic University, Hong Kong 999077, China; hu.hong@polyu.edu.hk

**Keywords:** displacement measurement, auxetic tubular structures, double-arrowhead auxetic structure, negative Poisson’s ratio

## Abstract

This research paper introduces an innovative technique for measuring displacement using auxetic tubular structure (ATS). The proposed displacement measurement method is based on tubular structures with a negative Poisson’s ratio. It capitalizes on the underlying principle that the elastic deformation-induced change in transmittance of the ATS can be translated into a corresponding modification in the output current of the solar cell. This method allows for the conversion of the variation in light transmission into a corresponding variation in output voltage. The construction of the ATS can be achieved through 3D-printing technology, enhancing the accessibility of displacement measurement and design flexibility. The experimental results demonstrate that the proposed measurement method exhibits a linear error of less than 8% without any subsequent signal processing and achieves a sensitivity of 0.011 V/mm without signal amplification. Furthermore, experimental results also show that the proposed method has good repeatability and can maintain a high level of reliability and sensitivity when using different measurement devices. This confirms the effectiveness and feasibility of the proposed method, showing a favorable linear relationship between the input and output of the measurement system with an acceptable sensitivity, repeatability, and reliability.

## 1. Introduction

Displacement refers to the alteration in the spatial location of an object or point, enabling us to comprehend the movement that has occurred. The determination of displacement gives engineers and scientists valuable information about the spatial dynamics of a system. The measurement of displacement plays a crucial role in areas such as the automotive industry, manufacturing processes, civil engineering, and aerospace industry. In essence, displacement measurement serves as a fundamental element of the engineering and industrial landscape. It enables meticulous control, monitoring, and assessment of diverse systems and processes, ensuring optimal performance, safety, and reliability. By accurately quantifying the spatial changes that occur, engineers and scientists can make informed decisions, and then troubleshoot problems.

Most displacement measurement schemes involve the conversion of displacement signals into electrical signals through displacement sensors. These sensors function as instruments utilized to measure the displacement or position of an object in relation to a reference point. These sensors operate based on various principles, encompassing resistive sensors [1,2,3], capacitive sensors [3], eddy current sensors [4,5,6], and optical fiber sensors [7,8,9,10]. Each sensor type possesses unique advantages and limitations, necessitating the consideration of factors such as desired accuracy, range, environmental conditions, and application-specific requirements when selecting an appropriate sensor.

Auxetic materials, often referred to as negative Poisson’s ratio materials [11,12,13], possess a distinctive attribute, expanding in all directions when subjected to stretching, and contracting in all directions when subject to compression [14,15,16]. This behavior stands in direct contrast to conventional materials. After the auxetic structure was first proposed by Evans in 1991, it has received widespread attention. In the following decades, researchers have discovered or designed various types of auxetic structures [17,18], such as arrowhead auxetic structures [19], chiral structures [20], 2D/3D re-entrant structures [21,22], sandwich structures [23,24,25], and so on. The reasons for different types of auxetic structures exhibiting negative Poisson’s ratio are varied. For example, the chiral unit cell flexibly folds due to the cylinders, whereas the neighboring unit cell undergoes vertical expansion as the re-entrant strut tends to migrate in the outward direction.

Auxetic materials and structures offer a range of intriguing possibilities and potential applications due to their unique properties. In the field of impact resistance [26], auxetic materials have been shown to absorb energy more effectively than conventional materials, making them ideal for use in protective equipment. Their flexibility and conformability also make them suitable for use in wearable technology and smart textiles [27,28,29]. In the biomedical field [30], auxetic materials have been investigated for use in bone implants, where their unique properties have been explored for their potential to enhance both the biological and mechanical performance of bone implants. Furthermore, sandwich structures with an auxetic core have potential applications in the aerospace field [31]. Auxetic materials and structures also have the potential for engineering measurement applications [32,33,34,35]. The unique properties of auxetic materials can be leveraged for accurate measurements of various physical quantities. For example, in force or pressure sensing applications, auxetic structures can be utilized to create sensors that exhibit changes in mechanical properties, such as stiffness or electrical conductivity, in response to external forces [36,37]. By incorporating auxetic materials into strain gauges or sensors, it is possible to achieve enhanced sensitivity and accuracy in measuring strain [38,39]. Introducing helical auxetic yarn into capacitive sensors benefits from their auxetic properties, enabling the sensors to achieve greater sensitivity under smaller strains [40].

Since the first negative Poisson’s tubular structure was developed [41], it has attracted a lot of attention [42,43,44]. There have been some researches working on its designed methods [45,46], and conducting characteristic and mechanism studies [47,48,49]. Due to their distinctive characteristics, there has been a growing interest in the study of ATSs. Weiwei [50] investigated the compressive behaviors of a series of ATSs by numerical and experimental methods, which showed that the negative Poisson’s ratio effect became obvious within a certain compression range. Lizhe [51] proposed and verified a nodal-coupling-based periodic boundary condition that could facilitate the parameterization study of the tubular lattice structure design. Plewa [52] designed a tubular structure based on the hinge mechanism and utilized rigid square frames as unit cells. This designed method provides us with a new approach when designing tubular structures. The results once again confirmed that the ATS exhibits a negative Poisson’s ratio when subjected to stretching.

This research paper introduces an innovative technique for measuring displacement using ATSs with a negative Poisson’s ratio. The method capitalizes on the principle that the elastic deformation-induced change in the transmittance of the structure can be translated into a corresponding modification in the output current of the solar cell. By exploiting the elastic deformation, the variation in light transmission is converted into a corresponding variation in output voltage. The construction of the ATS can be achieved through 3D-printing technology, enhancing the accessibility of displacement measurement and design flexibility. To evaluate the effectiveness of this proposed approach, the experimental investigations were conducted. The experimental results confirm the effectiveness, feasibility, repeatability, and reliability of the method, providing valuable insights into displacement sensors and laying the foundation for future studies in this area.

## 2. Structures and Methods

**Figure 1:**
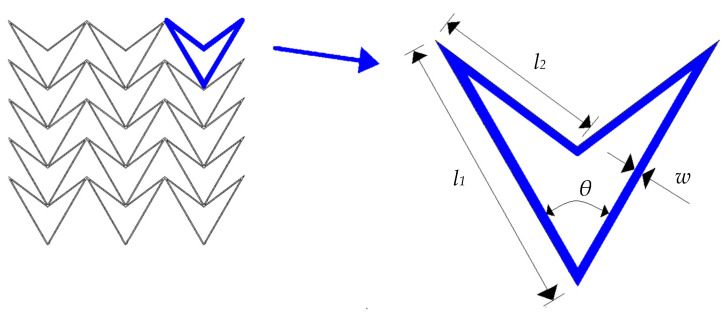
Geometry of 2D DA auxetic honeycomb structure and parameters of the unit cell.

As discussed in the literature review, there are many types of auxetic structures. While their characteristics may vary, they all possess the property of a negative Poisson’s ratio, leading to similar elastic deformation properties under compression. Consequently, the proposed method offers a range of options for auxetic structures. In this paper, the double-arrowhead (DA) structure is chosen as the unit cell. Figure 1 depicts the geometry of a two-dimensional (2D) DA auxetic honeycomb structure, which is formed by a specific arrangement of unit cells.

As shown in Figure 1, the cellular configuration of a DA auxetic honeycomb cell is defined by four important parameters. These parameters consist of the internal angles *θ* formed by the two inclined cell ribs, as well as the lengths (*l*_1_ and *l*_2_) of the corresponding inclined cell ribs, and the thickness *w* of the cell wall.

**Figure 2:**
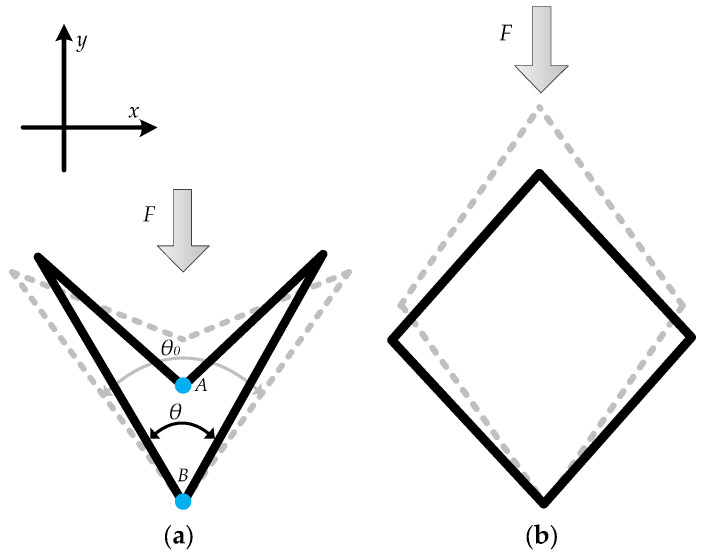
Deformation of the unit cell with (**a**) negative Poisson’s ratio and (**b**) positive Poisson’s ratio when compression force is applied.

The DA auxetic structure has a negative Poisson’s ratio due to its specific geometric arrangement and deformation mechanism. As shown in Figure 2, the indicated x-y coordinate system is a reference system that can help us conduct a qualitative analysis of the auxetic structure. When a compression force is applied to point A along the y-direction in Figure 2a, the inclined cell ribs undergo rotations and bending. This causes the unit cell to contract in the transverse direction and the length between points A and B to decrease, resulting in a negative Poisson’s ratio. Similarly, if a compression force is applied along the x-direction, the same tension and negative Poisson’s ratio would occur. However, this behavior is contrary to the lateral contraction observed in traditional materials when they are subjected to tension. If x- and y-directed compression forces are applied to the structure in Figure 2b, they would generate negative strains in the transverse direction, which indicates a positive Poisson’s ratio.

The Poisson’s ratio of the unit cell is given by Equation (1), which can be obtained based on [53].

(1) $$v_{yx}=-\frac{\varepsilon_{y}}{\varepsilon_{x}}=\frac{l_{12}\tan\frac{\theta }{2}\sqrt{2(1-\cos\theta )-{\left[l_{12}(1-\cos\theta )\right]}^{2}}}{l_{12}{}^{2}{\left(1-\cos\theta \right)}^{2}-2}$$

where $l_{12}=\frac{l_{1}}{l_{2}}$, and the definition of *l*_1_, *l*_2_ and *θ* are shown in Figure 1.

From Equation (1), it can be seen that the Poisson’s ratio is also determined by the four parameters mentioned above: *l*_1_, *l*_2_, *w*, and *θ*. Once a structure is designed, the values of *l*_1_, *l*_2_ and *w* remain constant. Therefore, when the structure undergoes stretching and deformation in the axial and transverse directions, the parameter that primarily changes within a certain range is *θ*.

**Figure 3:**
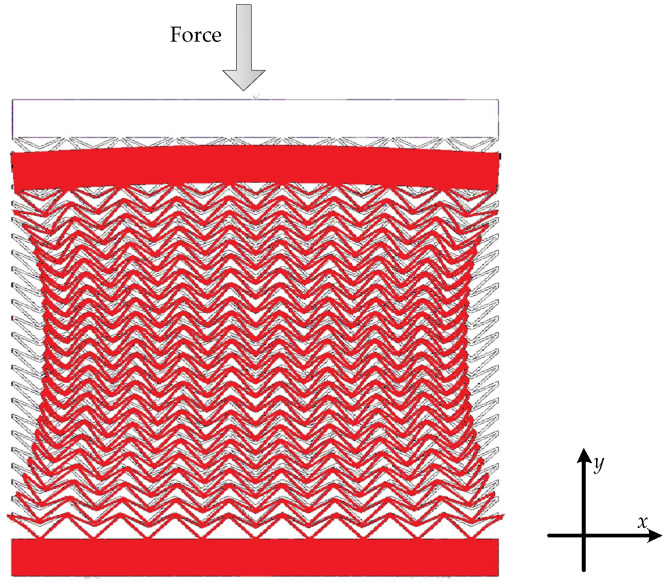
Deformation of the auxetic honeycomb structure.

A two-dimensional DA structure can be obtained by arranging the unit cells. As shown in Figure 3, when a compression force is applied along the y-direction, different unit cells at various positions in this structure exhibit different changes. The unit cells near the top and bottom of the structure, due to the different state from that in Figure 2, do not demonstrate a negative Poisson’s ratio. However, the unit cells in the middle region of the structure, subjected to compression force along the y-direction from upper unit cells, exhibit a negative Poisson’s ratio. Therefore, it can be observed from Figure 3 that after the structure is compressed in the y-direction, the middle region of the structure shows a significantly negative Poisson’s ratio, resulting in a noticeable contraction in the x-direction.

**Figure 4:**
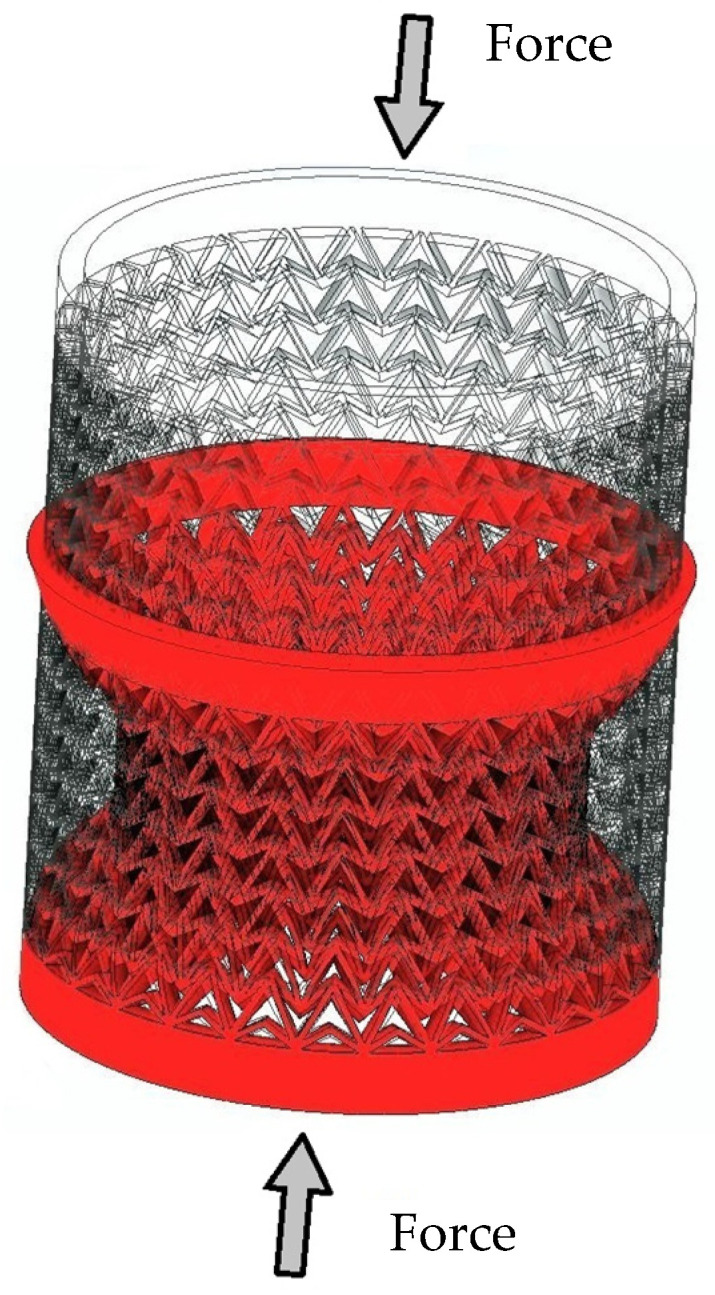
Deformation of the ATS.

As mentioned before, Plewa [52] designed an ATS by rolling up the planar structure of M×N unit cells. The theoretical values and measured data have consistently demonstrated that the ATS possesses a negative Poisson’s ratio of −1 since the linear expansion in both directions is the same. Based on the theory of Plewa, it is possible to extend the concept to a different type of structure. By transforming a two-dimensional DA honeycomb structure into a tubular form, composed of DA unit cells, a similar radial contraction deformation can be expected when the tubular structure is subjected to axial compression. This deformation can be visualized in Figure 4, where the tubular structure undergoes a radial contraction, resulting in a reduction in its diameter.

**Figure 5:**
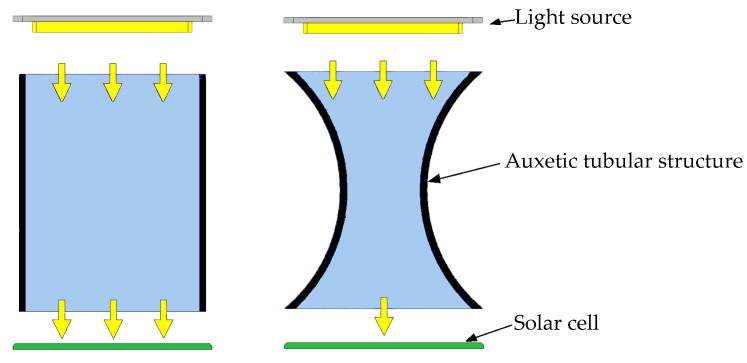
Principle of measurement method.

The displacement measurement method proposed in this paper is also based on the aforementioned characteristics of the tubular auxetic structure. Figure 5 shows the principle of the proposed measurement method. In this method, a planar light source is placed at one end of the axial direction of the tubular auxetic structure. The parallel light rays emitted by the light source pass through the tubular auxetic structure and illuminate the solar cell placed at the other end of the axial direction of the structure. The solar cell receives the light energy and converts it into electrical energy, generating a current. If the tubular auxetic structure undergoes deformation due to axial compression, it will experience radial contraction, causing some of the light rays to be obstructed and unable to reach the solar cell. This ultimately leads to a reduction in the current generated by the solar cell, as shown in Figure 5.

This measurement method is based on the characteristics of ATS, among which the regular and controllable deformation are the most important ones. Although the normal tubular structure can also show contraction when the axial compression force is applied, the deformation exhibits irregularity and uncontrollability. Thinvongpituk [54] studied the behavior of cylindrical shells under axial compression, which shows that the first indication of collapse occurs when an expanded axisymmetric ring forms near the bottom end during the collapse of the cylindrical shell. Therefore, the deformation exhibited by normal tubular structures under axial compression force cannot be effective in the proposed method. However, an ATS can be used because of the regular and controllable radial contraction when it is under the axial compression.

The radius of the ATS composed of *N* DA unit cells in the circumferential direction should follow the following pattern.

(2) $$r=\frac{Nl_{1}\sqrt{2\left(1-\cos\theta \right)}}{2\pi }$$

The area through which light can pass is given by

(3) $$S=\frac{{\left(Nl_{1}\right)}^{2}\left(1-\cos\theta \right)}{2\pi }$$

**Figure 6:**
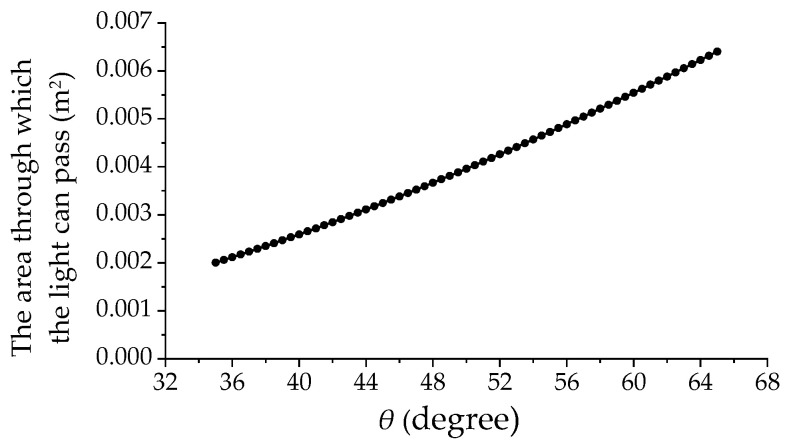
Variation of the area through which the light can pass with angle *θ*.

The size of the area through which light can pass determines the amount of light absorbed by the solar cell, which in turn determines the output of the solar cell. The current capacity of the solar cell is proportional to the intensity of light as well as the area that is exposed to the light. Figure 6 shows the relationship between the area and the angle *θ*. The figure is obtained when the ATS has the fixed dimensions of parameters that are shown in Table 1.

Based on Figure 6, it is evident that as the size of the light transmission area increases, the angle *θ* also increases, indicating an almost proportional relationship (Proportional Relationship A) between them. When a compression force is applied to the ATS along the y-direction, as shown in Figure 2a, the angle *θ* decreases accordingly. This decrease in angle leads to a reduction in the area of light passing through the structure, resulting in a decrease in the output of the solar cell. Therefore, by measuring the changes in the output of the solar cell, it becomes possible to accurately quantify the displacement or deformation of the structure. This relationship between the compression force that determines the displacement, angle *θ*, and the light transmission area is crucial for the operation of the proposed measurement method.

## 3. Experimental Study

### 3.1. Specimen Geometry

Re-entrant honeycomb structures, chiral structures, and rotating square structures are some of the well-known auxetic structures. Each of these structures has unique geometries and deformation mechanisms, resulting in different types of auxetic behavior. These structures have been extensively studied due to their potential applications in various fields. The DA structure is one of the popular designs in auxetic structures, thanks to its simplicity and effectiveness in achieving negative Poisson’s ratio behavior. Another reason for selecting the DA structure for this experiment is its predictable and controlled deformation behavior. This unique characteristic enables precise control over the structure’s response. The DA structure exhibits exceptional strength and stability, allowing it to withstand external forces while maintaining its structural integrity. This makes the DA structure a better choice for this study where strength stability is essential [55,56]. However, it should be further pointed out that other auxetic structures can also be used in this study. We choose the DA structure not only for the reasons mentioned above, but also simply because it is not necessary to specifically specify the use of a particular structure to achieve the objectives of this paper.

**Figure 7:**
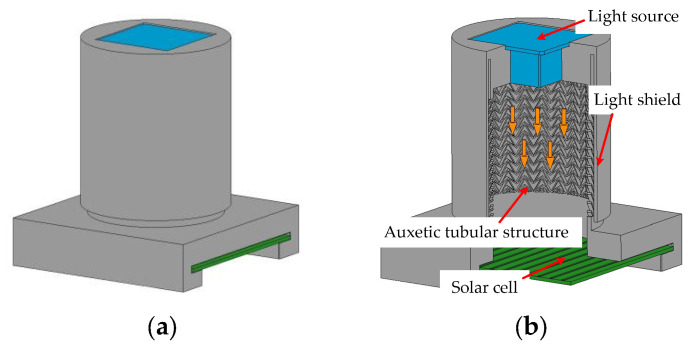
View of the measurement device based on ATS: (**a**) Full view; (**b**) 3/4 sectional view.

To further demonstrate the feasibility of the proposed displacement measurement method in this paper, the displacement measurement device was designed and manufactured. The basic structure of the device is shown in Figure 7. The main parts of the device are two installation holes and the ATS with a light shield (Figure 7b).

In a previous study [35], an auxetic structure for displacement measurement has been proposed. However, this method relies on changing the light transmittance of the honeycomb structure to measure displacement, making it susceptible to interference from external light. This limits its practical application in engineering. In this paper, we have designed a light shield on the outer surface of the ATS to mitigate this issue. The measurement apparatus comprises two separate layers. The outer layer is an outer cylindrical light shield that is used to reduce the impact of external light. The inner layer is the ATS. These two components are connected at the upper section, while the lower part of the light shield is suspended, ensuring that the entire applied load is exclusively supported by the ATS. At the top of the apparatus, there is an installation hole for the light source, while at the bottom section, another installation hole for the solar cell is designed.

As mentioned earlier, the DA structure has four structural parameters, namely *θ*, *l*_1_, *l*_2_ and *w*. For the purpose of this study, an auxetic structure is created using specific parameters that are shown in Table 2.

**Figure 8:**
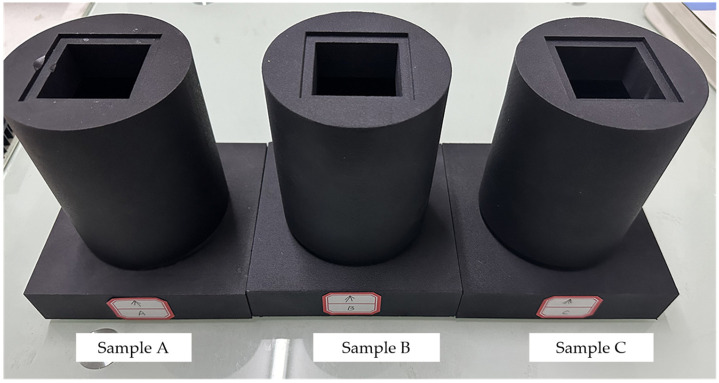
ATS specimens obtained by 3D printing.

The specimens used in this experiment, as depicted in Figure 8, are fabricated using a stereolithography (SLA) 3D printer. The elastic modulus of the material used to fabricate the specimen is 1.7 GPa, with a Poisson’s ratio of 0.4.

### 3.2. Experimental Set-Up

**Figure 9:**
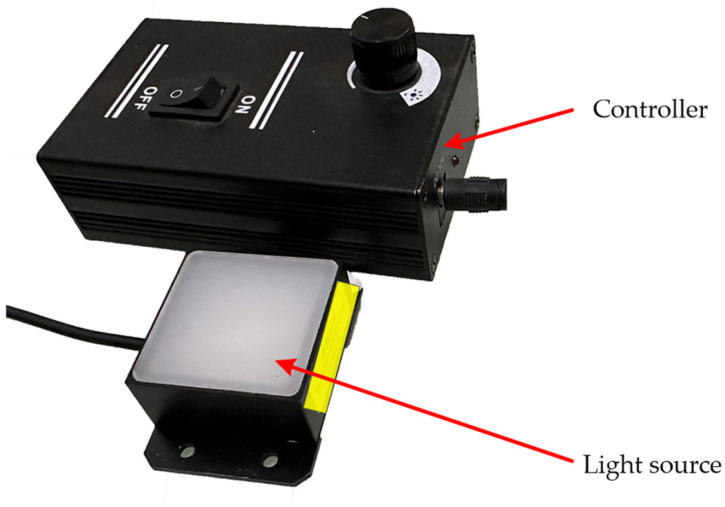
Light source and controller.

In this experiment, a planar light source (Figure 9) with an illuminated area of 40 × 40 mm^2^ was used as the experimental light source. The intensity of illumination from this light source can be measured as 360 Lux when the illuminance meter is placed 20 mm from the light source.

**Figure 10:**
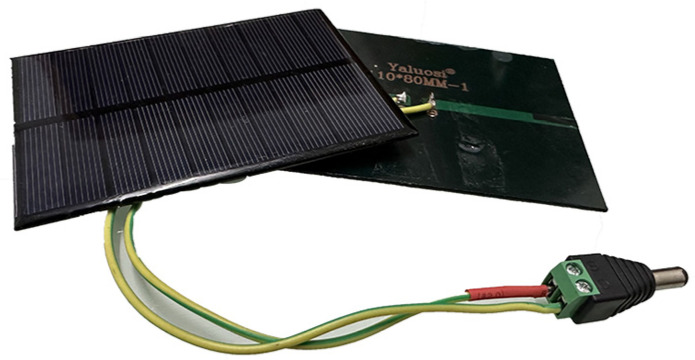
Single-crystal-silicon solar cell used in the experiment.

**Figure 11:**
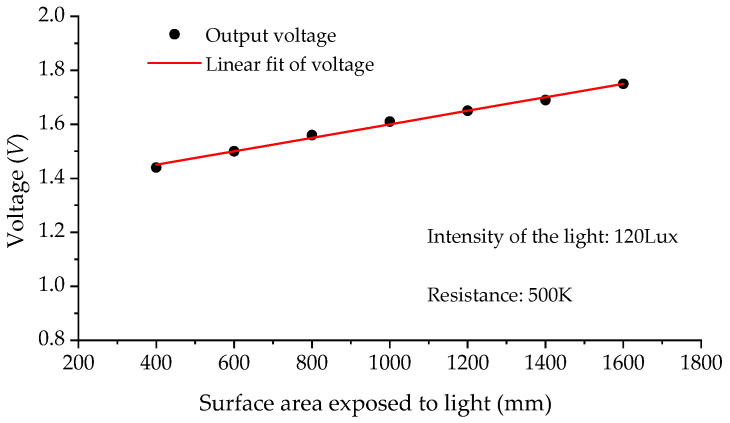
Relationship between output and exposed surface area of solar cell.

The role of the solar cell in this experiment is to convert the light energy incident on its surface into electrical energy. This study utilizes a single-crystal-silicon solar cell (see Figure 10, the parameters of the solar cell employed in this study are shown in Table 3.) to generate current output. The current generated by the solar cell is directly proportional to the intensity of the light incident on it and the exposed surface area (Proportional Relationship B, see Figure 11).

The two proportional relationships (Proportional Relationships A and B) mentioned above establish a direct correlation between the deformation of the ATS and the output of the solar cell. This implies that there is a definite relationship between the displacement and the output of the solar cell, which forms the fundamental principle of the measurement method proposed in this paper. The output of the solar cell accurately reflects the structural deformation, which is precisely the displacement measured by the proposed method.

**Figure 12:**
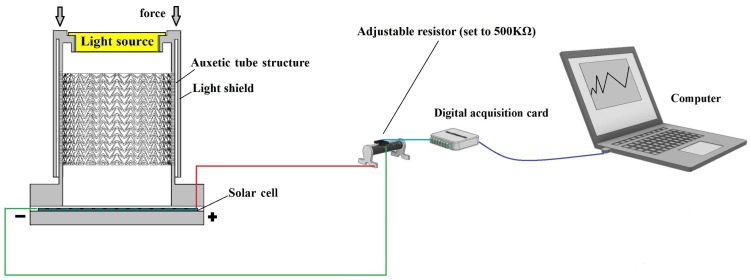
Schematic diagram of the experimental set-up.

To facilitate the investigation, a resistor is connected in series with the solar cell. The generated current by the solar cell is directly proportional to the prevailing light intensity, causing corresponding changes in the voltage across the resistor. Therefore, monitoring the voltage variations across the resistor provides a reliable indicator for determining the level of light intensity. This setup allows for convenient measurement and analysis of the solar cell’s response to different light conditions. For a visual representation of the experimental setup, please refer to Figure 12.

**Figure 13:**
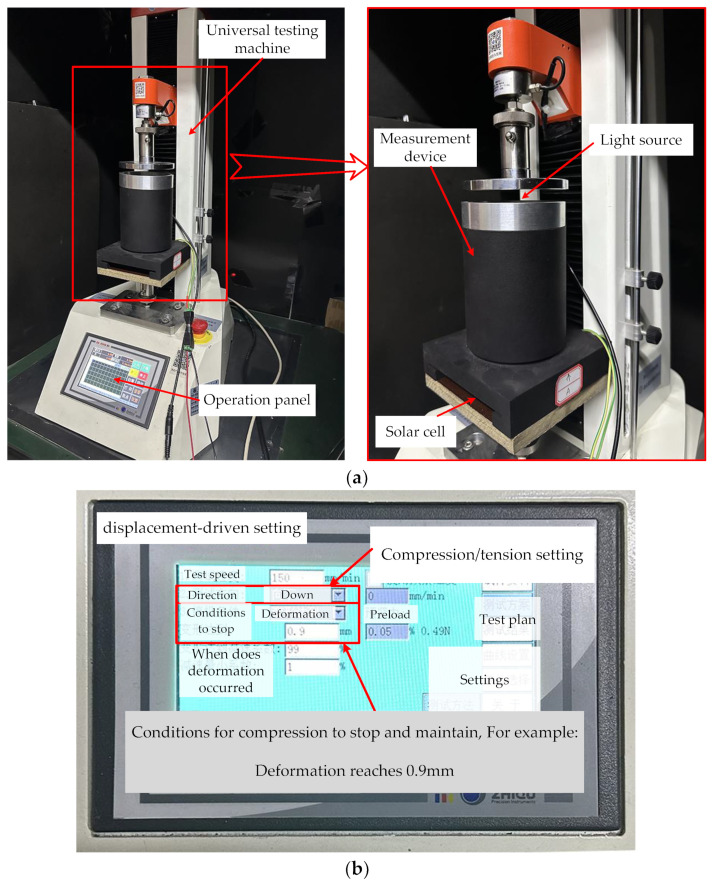
Test rig and setting mode: (**a**) universal testing machine and the ATS sample, (**b**) example of displacement-driven setting.

The test rig is shown in Figure 13. The use of a universal testing machine (Figure 13a) in this experiment allows for precise displacement control of the ATS. The universal testing machine is programmed to apply a predetermined displacement by compressing an ATS and maintaining it for a certain duration. A maximum displacement of 2 mm was predetermined and divided into 20 increments, which means that the universal testing machine applies incremental displacements of 0.1 mm to the ATS (Figure 13b). After each displacement increment, the output voltage of the solar cell is then recorded. This allows for the measurement and monitoring of the changes in the output voltage, which corresponds to the deformation or displacement of the ATS.

To better study the deformation process of the ATS under compression, the entire experiment was divided into two parts: gradual increase in compression and gradual decrease in compression. In other words, the first step involves compressing by 0.1 mm, and then increasing the compression by 0.1 mm for each subsequent step until reaching the maximum compression of 2 mm. Then, the compression is reduced to 1.9 mm, followed by further reductions of 1.8 mm, and so on, until the compression reaches 0 mm.

The use of a data acquisition card in this experiment allows for the accurate and reliable collection of data. The JYTEK USB-61210 data acquisition card (16-bit, 4 differential, 2 MS/s per channel) can collect high-resolution data at a sampling rate of 10 KHz, enabling precise measurement of the data. The LabView software used for data acquisition provides a user-friendly interface for controlling the data acquisition card and collecting data. By programming the software to record the average value of data collected for 10 s, the accuracy and reliability of the data are further improved. To ensure experimental stability and consistency, a continuous running time of 1 min is allowed before data acquisition during the experimental procedure.

To validate the validity of the proposed method, three experiments are conducted. The initial experiment employs sample A as the ATS, with the primary objective being to showcase the high sensitivity and linearity of the proposed measurement method. In the subsequent experiment, sample B is employed, where a total of 5 experiments were executed to exhibit the repeatability of the method. Lastly, all three structures (Sample A, B, and C) were utilized in the third experiment to demonstrate the displacement measurement method’s stability when applied to different structures with identical parameters.

## 4. Results and Discussion

In the absence of an externally applied force, the initial output voltage of the solar cell can be denoted as *V*_0_. However, upon the application of a force, the structure undergoes a contraction process, resulting in an increase in light obstruction per unit area and subsequently causing a reduction in the output current of the solar cell. This phenomenon induces variations in the output voltage, denoted as *V_output_*. The magnitude of voltage change (Δ*V*) can be defined as

(4) $$\Delta V=V_{output}-V_{0}$$

**Figure 14:**
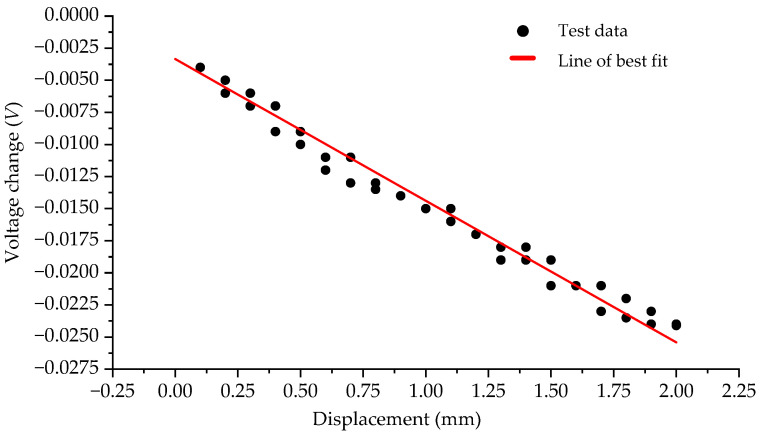
Relationship between the voltage change and the displacement when sample A is used in the experiment.

The relationship between the displacement of ATS (Sample A) and the voltage output of the solar cell is depicted in Figure 14. To further comprehend the displacement-voltage relationship, a line of best fit has been generated using the Least Square Method and is represented by a red line in Figure 14. The slope of the line of best fit serves as a quantitative measure of the sensitivity of the proposed displacement measurement method and is a key parameter in evaluating the performance and accuracy of the proposed method.

From Figure 14, it is evident that the proposed measurement method demonstrates good linearity overall. However, significant errors (up to 12%) are observed at smaller displacement values, particularly close to zero. If the errors near the zero value are disregarded, the linear error does not exceed 8%. It is important to note that these results are obtained without any signal processing. Additionally, Figure 14 indicates a sensitivity of 0.011 V/mm for the measurement method, which is also obtained without any signal processing or amplification. It is reasonable to believe that the proposed measurement method can achieve improved performance through the incorporation of signal processing techniques. Furthermore, in the proposed measurement method, the sensitivity is primarily determined by Poisson’s ratio of the measurement structure, which is influenced by the four parameters of the unit cell structure. By optimizing the design of the unit cell structure, it is possible to further improve the sensitivity of the measurement method. Through structural optimization, it is possible to adjust the parameters of the unit cell structure to achieve an appropriate Poisson’s ratio. This, in turn, can enhance the sensitivity of the measurement method, making it more responsive to small displacements and improving the accuracy of the measurements. By reducing errors and increasing sensitivity, the measurement method can be further refined and optimized for more accurate and reliable displacement measurements.

**Figure 15:**
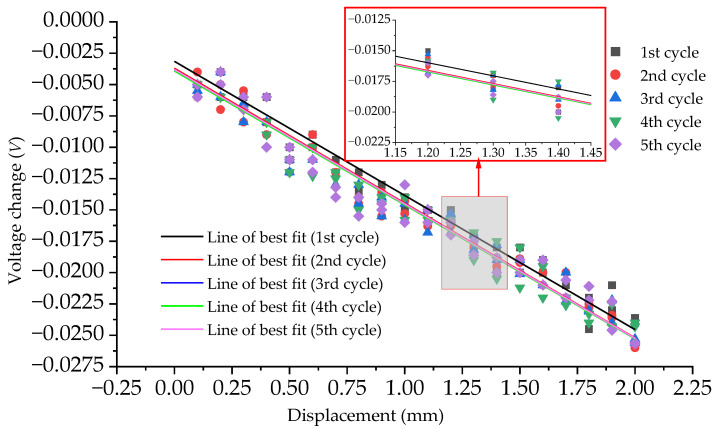
Relationships between the voltage change and the displacement of each experiment when sample B is used in all 5 experiments.

The experiment was conducted a total of five times, using sample B as the measurement device and under identical testing conditions. Each experiment was conducted approximately 30 min apart. The experimental procedure remained consistent throughout all five experiments, starting from 0 mm and testing every 0.1 mm until 2 mm, then decreasing by 0.1 mm each time until reaching 0 mm. The results of these five experiments can be found in Figure 15. It can be concluded that the method proposed in this paper has good repeatability. The slope of each line of the best fit curve obtained from the experimental results using sample B is a measure of the sensitivity of the proposed method. Table 4 shows that the sensitivities of the five experiments are very similar, with only minor variations observed. The good repeatability and minor variations in sensitivity indicate that the method can be reliably replicated and applied consistently. This suggests that the proposed method is robust and can be trusted for accurate and consistent results.

The method proposed in this paper comprises three key components: the measurement device, the light source, and the solar cell. A notable advantage of this method is the independence of these three parts, which allows for easy replacement or interchangeability. Firstly, it provides flexibility in terms of component selection and customization. Researchers or practitioners can choose different measurement devices, light sources, or solar cells following some instructions based on their specific requirements or preferences. This allows for optimization of the system based on factors such as accuracy, sensitivity, or availability of components. Secondly, the independence of these components enables easy maintenance and troubleshooting. If any component malfunctions or requires repair, it can be replaced individually without affecting the functionality of the entire system. This reduces downtime and increases the overall reliability of the measurement method. Additionally, the independence of the components allows for future upgrades or advancements. However, assuming that the light source and solar cell remain unchanged, whether replacing different structures with the same size and material would affect the measurement results should be considered.

**Figure 16:**
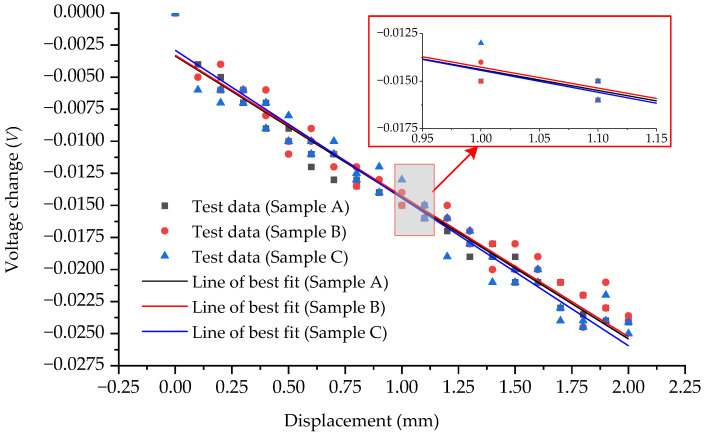
Relationships between the voltage change and the displacement when all three samples are used in the experiment.

Figure 16 shows the relationships between the voltage change and the displacement when using all three samples in the experiment. Table 5 shows the sensitivities of the measurement method using all three samples. It is evident that when using three samples of the same size and material as the measurement device, the results obtained are consistently close. This suggests that the method can maintain a high level of reliability and sensitivity when replacing the measuring device.

The close agreement between the results obtained using different samples further supports the notion that the method is robust and dependable. It implies that the measurement device itself does not introduce significant variations in the measurements. As a result, the method can be considered reliable and sensitive, ensuring accurate and consistent results. The findings from using three samples of the same size and material as the measurement device suggest that this method can effectively maintain good reliability and sensitivity when replacing the measuring device. This is an important aspect to consider in practical applications, where the need for device replacement or interchangeability may arise without compromising the accuracy and consistency of the measurements.

## 5. Conclusions

This paper proposed a displacement measurement method based on the ATS. This method consists of a tubular structure with a negative Poisson’s ratio, a light source, and a solar cell. Its effectiveness has been demonstrated through the development of a straightforward measurement system, which establishes a linear relationship between the displacement and the resulting electrical signal. The experimental result obtained without any signal processing or amplification enabled the authors to draw the following conclusions:

1. The proposed displacement measurement method using ATSs and solar cells demonstrates good linearity overall and the linear error does not exceed 8%.
2. The sensitivity of the proposed method is determined to be 0.011 V/mm. This sensitivity can be further improved by optimizing the design of the DA ATS.
3. Good repeatability with minor variations in sensitivity indicates that the proposed method is robust.
4. The close agreement between the experimental results obtained using different samples as measurement devices suggests that the proposed method can maintain a high level of reliability and sensitivity when replacing the measurement device, and further supports the robustness and dependability.
5. The proposed measurement method offers the advantage of independence between the measurement device, light source, and solar cell. This allows for easy replacement or interchangeability, customization based on specific requirements, and simplified maintenance.

This investigation provides novel perspectives and opportunities for the advancement of displacement measurement techniques. The simplicity of our proposed method offers advantages in terms of accessibility and affordability, which may facilitate its widespread adoption in various industries and applications.

## Figures and Tables

**Table 1:** Parameters of the auxetic structure used to determine the relationship between area and *θ*.

Parameter Name	Value	Unit
*θ*	35–65	degree
*l*_1_	5.5	mm
*l*_2_	4.5	mm
*w*	0.5	mm
*N*	48	/

**Table 2:** Parameters of the auxetic structure used to fabricate the measurement devices.

Parameter Name	Value	Unit
*θ*	60	degree
*l*_1_	5.5	mm
*l*_2_	4.5	mm
*w*	0.5	mm

**Table 3:** Parameters of solar cell.

Parameter Name	Value	Unit
Length	110	mm
Width	80	mm
Conversion efficiency	20%	/

**Table 4:** Sensitivities of the measurement method using sample B in 5 experiments.

Experiment Order	Sensitivity	Unit
1st	0.01088	V/mm
2nd	0.01085	V/mm
3rd	0.01089	V/mm
4th	0.01086	V/mm
5th	0.01083	V/mm

**Table 5:** Sensitivities of the measurement method using all three samples.

Sample	Sensitivity	Unit
A	0.01103	V/mm
B	0.01088	V/mm
C	0.01112	V/mm

## Data Availability

The data that support the findings of this study are openly available in https://github.com/NewHost/Data-for-Sensors, accessed on 10 November 2023.
